# Atypical Posterior Sympathetic Ophthalmia Shortly After PRESERFLO™ MicroShunt Implantation Masquerading as Hypotony Maculopathy: A Case Report

**DOI:** 10.7759/cureus.97220

**Published:** 2025-11-19

**Authors:** Kenji Fukuzawa, Hiroki Mieno, Kenji Nagata, Morio Ueno, Chie Sotozono

**Affiliations:** 1 Department of Ophthalmology, Machida Hospital, Kochi, JPN; 2 Department of Ophthalmology, Kyoto Prefectural University of Medicine, Kyoto, JPN; 3 Department of Ophthalmology, Nagata Eye Clinic, Kyoto, JPN

**Keywords:** glaucoma surgery, hypotony maculopathy, posterior variant, preserflo™ microshunt, sympathetic ophthalmia

## Abstract

We report a rare case of presumed posterior sympathetic ophthalmia (SO) occurring shortly after PRESERFLO™ MicroShunt (PMS) (Santen Pharmaceutical Co., Ltd., Osaka, Japan) implantation that initially mimicked hypotony maculopathy. A 42-year-old woman with a history of multiple prior intraocular surgeries underwent PMS implantation in her left eye for glaucoma with uncontrolled intraocular pressure (IOP). At three days post-surgery, visual acuity (VA) decreased to hand motion, and IOP dropped to 6 mmHg. Slit-lamp examination showed no keratic precipitates or anterior chamber inflammation, while fundoscopy imaging revealed choroidal folds and serous retinal detachment. Given a provisional diagnosis of hypotony maculopathy, an anterior chamber air injection was performed at six days post-surgery. Despite a resultant rise in IOP, the posterior findings did not improve. Thus, a diagnosis of SO was made, and high-dose intravenous corticosteroid pulse therapy followed by oral corticosteroids was initiated at 10 days post-surgery, which led to anatomic improvement. Human leukocyte antigen (HLA) typing was positive for HLA-DR4. At two years post-surgery, her left eye had a decimal VA of 0.2 and a stable IOP of 10 mmHg. Oral prednisolone was tapered to 5 mg/day without recurrence. Fundoscopy examination of her right eye was difficult due to long-standing total retinal detachment. This case underscores several atypical features of posterior SO, including very early-onset, posterior-predominant disease without anterior inflammation, and diagnostic challenges in the fellow eye, and highlights the importance of considering SO in atypical postoperative presentations, even after less invasive glaucoma surgery, and the need for early recognition with timely corticosteroid therapy to preserve vision.

## Introduction

Sympathetic ophthalmia (SO) is a rare, sight-threatening, bilateral granulomatous panuveitis that classically occurs after penetrating ocular trauma or intraocular surgery, presumably due to loss of ocular immune privilege and autoimmune reactivity against ocular antigens [1-3]. Clinically, SO is characterized by bilateral granulomatous anterior uveitis with mutton-fat keratic precipitates and iris nodules, uveitis, and posterior pole-predominant multifocal yellow-white chorioretinal lesions accompanied by serous retinal detachment and optic disc hyperemia. Although SO usually presents with bilateral granulomatous panuveitis, an atypical form with only posterior segment findings has been identified and termed as posterior SO [4,5]. SO has been reported following a wide spectrum of procedures, including penetrating keratoplasty, cyclodestructive procedures, cataract, vitreoretinal, and glaucoma surgeries [6].

The PRESERFLO™ MicroShunt (PMS; Santen Pharmaceutical Co., Ltd., Osaka, Japan) is a novel device composed of a biocompatible and bioinert synthetic polymer material (i.e., poly(styrene-block-isobutylene-block-styrene), also known as "SIBS") that is designed to provide significant intraocular pressure (IOP)-lowering effects while reducing many surgical risks and postoperative management requirements associated with trabeculectomy and tube shunt implantation [7-10]. However, postoperative hypotony is a well-recognized complication associated with PMS implantation, with incidences reportedly ranging from 1.7% to 39% [11]. Clinically, postoperative hypotony may manifest as hypotony maculopathy, which is characterized by radiating chorioretinal folds involving the posterior pole, peripheral serous choroidal detachments/effusions, and variable optic disc hyperemia or mild edema with vascular tortuosity [12,13].

Herein, we report a case of posterior SO occurring shortly after PMS implantation that initially mimicked hypotony maculopathy.

## Case presentation

A 42-year-old woman with a history of atopic dermatitis was referred to the Department of Ophthalmology at Kyoto Prefectural University of Medicine, Kyoto, Japan, due to persistent IOP elevation in her left eye. Four months before her initial presentation, Graves' disease with thyrotoxicosis was diagnosed, and antithyroid therapy was initiated at another hospital. Around the same time, she experienced ocular pain and presented to the Department of Ophthalmology of that hospital, where her IOP was found to be 37 mmHg OD and 44 mmHg OS. Although she had previously undergone ocular surgeries, she had discontinued undergoing follow-up examinations until the current presentation. Notably, she had undergone two pars plana vitrectomies in her left eye, one for rhegmatogenous retinal detachment (RRD) and one for intraocular lens dislocation, and a vitrectomy for RRD with initial anatomic reattachment in her right eye. However, after she discontinued follow-up examinations, her right eye progressed to long-standing total retinal detachment with no light perception. Her left eye was diagnosed with secondary glaucoma following multiple prior intraocular surgeries related to atopic dermatitis-associated ocular disease. In contrast, the right eye exhibited iris neovascularization with complete angle closure, consistent with neovascular glaucoma-related intraocular pressure elevation. Since IOP control remained inadequate on maximally tolerated topical therapy, micropulse transscleral cyclophotocoagulation (MP-TSCPC) was performed once in her left eye and twice in her right eye. However, IOP remained uncontrolled after MP-TSCPC, so she was referred to our department two months later for surgical management of the left eye, which was her only eye with vision, as her right eye had no light perception.

**Figure 1:**
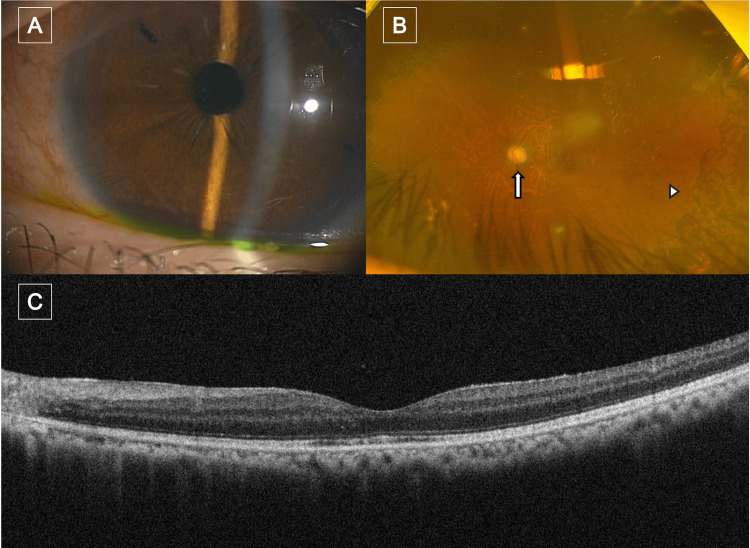
Images of the patient's left eye at initial presentation (A) Slit-lamp photograph showing a scleral-fixated intraocular lens with no anterior segment inflammation. (B) Fundus photograph demonstrating glaucomatous optic neuropathy (arrow) and peripheral retinal atrophy (arrowhead), likely secondary to prior laser photocoagulation for retinal detachment. (C) Spectral domain OCT image of the macula showing a preserved foveal contour without abnormalities. OCT: optical coherence tomography

**Figure 2:**
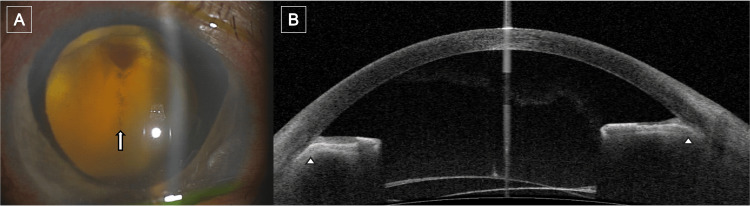
Images of the patient's right eye at initial presentation (A) Slit-lamp photograph showing total retinal detachment (arrow). (B) Anterior segment OCT image demonstrating complete angle closure with peripheral anterior synechiae between the iris and trabecular meshwork (arrowheads). OCT: optical coherence tomography

At presentation, best-corrected visual acuity (BCVA) in the left eye was 0.6 (decimal), and slit-lamp examination showed a scleral-fixated intraocular lens with no anterior segment abnormalities. Fundoscopy examination revealed glaucomatous optic neuropathy and peripheral retinal atrophy, which were likely secondary to prior laser photocoagulation for retinal detachment (Figure 1). Her right eye was found to have total retinal detachment with complete angle closure and no apparent iris neovascularization (Figure 2). Despite the administration of five antiglaucoma agents, the IOP in her right and left eyes were 34 mmHg and 31 mmHg, respectively. Hence, PMS implantation surgery was performed in her left eye with no intraoperative complications, and topical administration of corticosteroid and antibacterial eye drops was initiated post-surgery.

**Figure 3:**
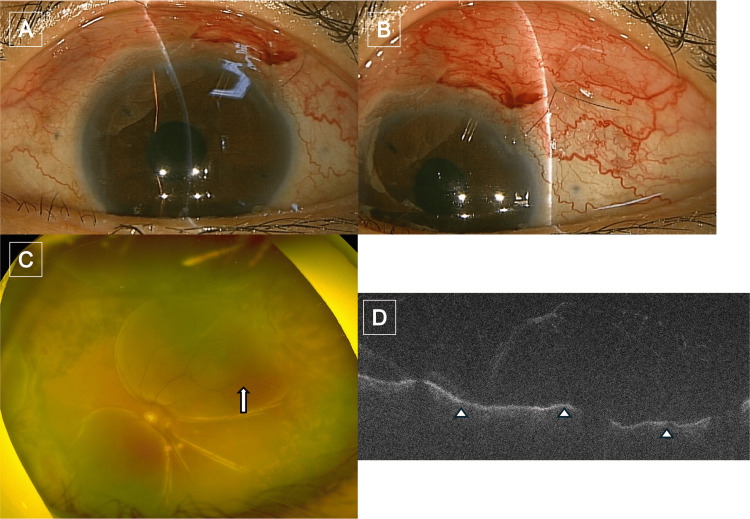
Images of the patient's left eye at the onset of sympathetic ophthalmia (A) Slit-lamp photograph showing no keratic precipitates or anterior segment inflammation. (B) Slit-lamp photograph of the filtering bleb without abnormal findings. (C) Fundus photograph demonstrating choroidal folds and serous retinal detachment (arrow). (D) Swept-source OCT image of the macula showing undulating changes of the retinal pigment epithelium and serous retinal detachment (arrowheads). OCT: optical coherence tomography

**Figure 4:**
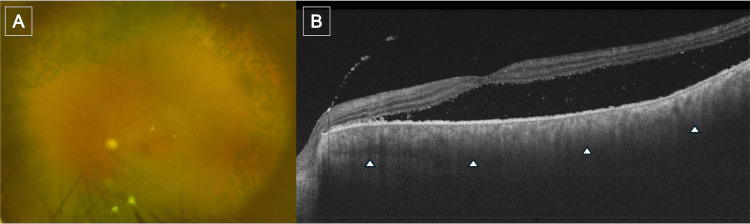
Findings obtained prior to the second high-dose intravenous corticosteroid pulse therapy (A) Fundus photograph showing reduced choroidal folds and serous retinal detachment compared with the onset. (B) Swept-source OCT image of the macula showing decreased serous retinal detachment with improved retinal pigment epithelium undulation, yet persistent choroidal thickening with poorly defined vascular lumina (arrowheads). OCT: optical coherence tomography

**Figure 5:**
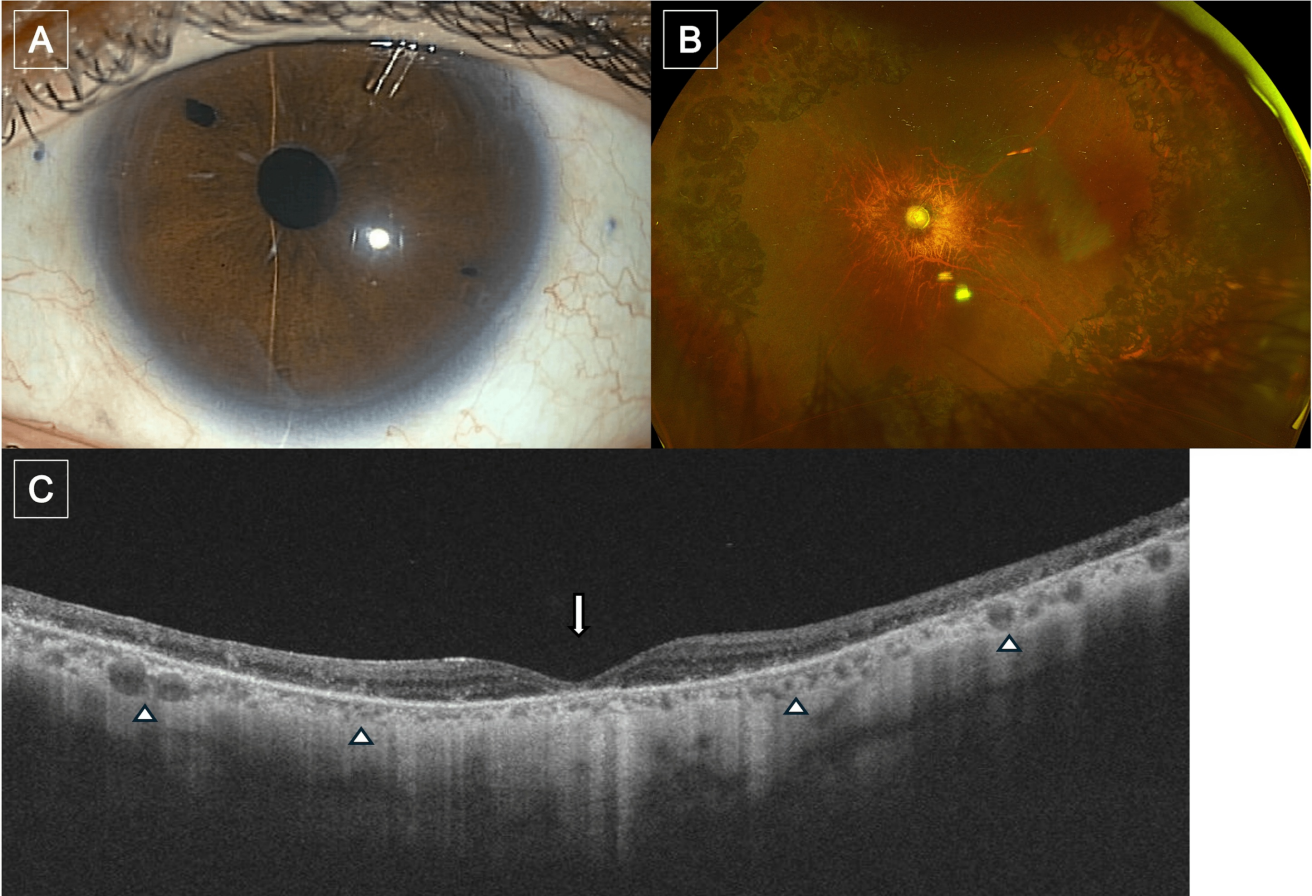
Ocular findings of the left eye at two years after onset (A) Slit-lamp photograph showing a quiet anterior segment with the PRESERFLO™ MicroShunt visible. (B) Fundus photograph showing resolution of serous retinal detachment with atrophic changes in the macular region. (C) Swept-source OCT image of the macula demonstrating atrophic changes (arrow), with no choroidal thickening and clearly delineated choroidal vascular lumina (arrowheads). OCT: optical coherence tomography

Three days postoperatively, vision in her left eye declined to hand motion, and IOP dropped to 6 mmHg. Slit-lamp biomicroscopy demonstrated a clear cornea without keratic precipitates and a deep and mostly quiet anterior chamber with no significant cells or flare, an appropriately positioned PMS tube, and no evidence of bleb hyperfiltration. Fundus examination revealed choroidal folds and serous retinal detachment. Swept-source optical coherence tomography (OCT) imaging of the macula showed undulating changes in the retinal pigment epithelium (RPE) and serous retinal detachment (Figure 3). Since the provisional diagnosis was hypotony maculopathy, an anterior chamber air injection was performed six days postoperatively. Despite IOP elevation after the anterior chamber air injection, the posterior findings did not improve, so a diagnosis of SO was made, and high-dose intravenous corticosteroid pulse therapy (500 mg/day, three days) was initiated four days later. Given the favorable anatomic response (Figure 4), a second three-day pulse was administered, and oral prednisolone (50 mg/day) was initiated. Swept-source OCT imaging of the macula showed decreased serous retinal detachment with improved RPE undulation. However, persistent choroidal thickening with poorly defined vascular lumina was observed, although no choroidal detachment was noted. Due to subsequent IOP elevation, filtration bleb revision was performed one month postoperatively. A transient exacerbation of choroidal effusions occurred after IOP reduction, followed by gradual resolution. By five months postoperatively, both choroidal thickening and serous retinal detachment had completely resolved. Human leukocyte antigen (HLA) typing was positive for HLA-DR4. At two years postoperatively, BCVA in her left eye was 0.2 (decimal) and IOP in that eye was stable at 10 mmHg (Figure 5), so oral prednisolone was then tapered to 5 mg/day without recurrence.

## Discussion

We report a rare case of posterior SO that developed shortly after PMS implantation and initially masqueraded as hypotony maculopathy. The incidence proportion of SO after glaucoma surgery has recently been estimated at 0.098% [1]. However, to the best of our knowledge, this represents the first reported case of SO following PMS implantation. The patient's HLA-DR4 positivity, previously linked with an increased susceptibility to SO [14], suggests an underlying immunogenetic predisposition.

Before discussing the atypical features of this case, Vogt-Koyanagi-Harada (VKH) disease was considered as the primary differential diagnosis, as it shares several clinical characteristics with SO, including bilateral involvement, serous retinal detachment, choroidal thickening, and HLA-DR4 positivity [3]. However, VKH was excluded because there was a clear history of intraocular surgery serving as an inciting event, an essential criterion for SO, and no systemic manifestations such as meningismus, vitiligo, alopecia, or hearing disturbance were present.

The present case exhibited several atypical features compared with the usual manifestations of SO, which thus merit detailed consideration.

First, the onset was remarkably early. SO usually develops within five days to six months after ocular trauma or intraocular surgery [1], with the majority of cases occurring within the first year [15]. In our patient, the disease rapidly occurred three days after PMS implantation, which is early. Although rare, a diagnosis as early as five days after penetrating ocular trauma has been reported [16], underscoring that while extraordinary, the early presentation in our patient falls within the spectrum of very early manifestations presented in the published literature.

We suggest that the exceptionally early onset in our patient may reflect cumulative immune sensitization from multiple prior intraocular procedures. Repeated inciting events have been reported as a major risk factor for the onset of SO [17,18]. In our case, both eyes had undergone pars plana vitrectomy, making it difficult to definitively identify a single inciting eye. However, because no signs of SO were present before PMS implantation, the PMS surgery was considered the most likely final inciting event following cumulative sensitization.

Second, the inflammatory pattern in the inciting eye was atypical. While SO classically presents with granulomatous anterior uveitis, keratic precipitates, and diffuse panuveitis, our patient exhibited only posterior segment changes, serous retinal detachment and choroidal folds, and choroidal thickening with poorly defined vascular lumina without detectable keratic precipitates or significant anterior chamber inflammation. This constellation of findings is consistent with the so-called posterior variant of SO, in which posterior segment involvement dominates and anterior segment inflammation is minimal or absent at onset [4,5]. Our patient was in the immediate postoperative period, and the concomitant use of topical corticosteroids may have contributed to the absence of anterior segment inflammation at the time of onset.

Third, the fellow right eye was difficult to examine for detailed fundus changes due to long-standing total retinal detachment. Hence, it was difficult to detect findings suggestive of SO observed on the left eye, which contributed to the delayed diagnosis.

Taken together, these features, i.e., an exceptionally early onset, posterior-predominant findings with no anterior inflammation, and diagnostic challenges in the fellow eye, made it particularly challenging to distinguish the presentation from hypotony maculopathy. The posterior segment abnormalities persisted despite normalization of intraocular pressure, which is inconsistent with hypotony maculopathy and instead supports a diagnosis of SO. In the initial cases that underwent PMS implantation, postoperative hypotony was relatively frequent, yet more recently, placement of an intraluminal stent has been reported to effectively prevent early hypotony [19,20]. Wider adoption of this technique may reduce postoperative hypotony and subsequently facilitate differentiation from other entities.

## Conclusions

The findings in this case, including exceptionally early onset, posterior-predominant involvement with no anterior inflammation, and limited fellow eye assessment marked by a paradoxical IOP shift, indicate posterior sympathetic ophthalmia shortly after PMS implantation, initially masquerading as hypotony maculopathy. These features underscore the need to consider SO in atypical postoperative courses even after less-invasive glaucoma procedures, as early diagnosis and prompt corticosteroid therapy are critical to optimize anatomic resolution and preserve vision.
